# Systematic review of interventions in the childcare setting with direct parental involvement: effectiveness on child weight status and energy balance-related behaviours

**DOI:** 10.1186/s12966-019-0874-6

**Published:** 2019-11-21

**Authors:** I. van de Kolk, S. R. B. Verjans-Janssen, J. S. Gubbels, S. P. J. Kremers, S. M. P. L. Gerards

**Affiliations:** Department of Health Promotion, School of Nutrition and Translational Research in Metabolism (NUTRIM), Maastricht University, PO Box 616, 6200 MD Maastricht, the Netherlands

**Keywords:** Childcare, Effectiveness, Interventions, Nutrition, Parental involvement, Physical activity, Preschool, Sedentary behaviour, Weight status

## Abstract

**Background:**

The early years are a crucial period to promote healthy energy balance-related behaviours in children and prevent overweight and obesity. The childcare setting is important for health-promoting interventions. Increasingly, attention has been paid to parental involvement in childcare-based interventions. The aim of this systematic review is to evaluate the effectiveness of these interventions with direct parental involvement on the children’s weight status and behavioural outcomes.

**Methods:**

A systematic search was conducted in four electronic databases to include studies up until January 2019. Studies written in English, describing results on relevant outcomes (weight status, physical activity, sedentary behaviour and/or nutrition-related behaviour) of childcare-based interventions with direct parental involvement were included. Studies not adopting a pre-post-test design or reporting on pilot studies were excluded. To improve comparability, effect sizes (Cohen’s d) were calculated. Information on different types of environment targeted (e.g., social, physical, political and economic) was extracted in order to narratively examine potential working principles of effective interventions.

**Results:**

A total of 22 studies, describing 17 different interventions, were included. With regard to the intervention group, 61.1% found some favourable results on weight status, 73.3% on physical activity, 88.9% on sedentary behaviour, and all on nutrition-related behaviour. There were studies that also showed unfavourable results. Only a small number of studies was able to show significant differences between the intervention and control group (22.2% weight status, 60.0% physical activity, 66.6% sedentary behaviour, 76.9% nutrition behaviour). Effect sizes, if available, were predominantly small to moderate, with some exceptions with large effect sizes. The interventions predominantly targeted the socio-cultural and physical environments in both the childcare and home settings. Including changes in the political environment in the intervention and a higher level of intensity of parental involvement appeared to positively impact intervention effectiveness.

**Conclusion:**

Childcare-based interventions with direct parental involvement show promising effects on the children’s energy balance-related behaviours. However, evidence on effectiveness is limited, particularly for weight-related outcomes. Better understanding of how to reach and involve parents may be essential for strengthening intervention effectiveness.

## Background

In the past decades, the prevalence of childhood overweight and obesity has increased dramatically, and although a plateauing of the prevalence can be seen [1], their prevention remains an important issue in public health. Research on childhood overweight and obesity has shown that weight status in young children (age 2–6 years old) is most predictive for weight status as adults [2, 3].

One cause of overweight and obesity is a disruption in the body’s energy balance [4]. Promoting healthy energy balance-related behaviours (EBRBs), such as the consumption of fruit and vegetables, higher levels of daily physical activity and low levels of sedentary behaviour (e.g. television viewing), is important to prevent childhood overweight and obesity [5, 6]. It is known that overweight-related lifestyle behaviours track from childhood into adulthood, just like weight status [7]. Therefore, early childhood provides a window of opportunity for the prevention of overweight and obesity [8].

EBRBs are influenced by multiple factors, such as the child’s environment [9]. From a socio-ecological perspective, different types of environments and different settings can influence behaviour [9–11]. Environments can be categorized into sociocultural (attitudes, beliefs and values related to nutrition and physical activity within a setting); physical (what is available); economic (costs related to nutrition and physical activity); and political (rules, regulations, policies, and laws related to nutrition and physical activity) [11].

One setting that influences children’s EBRBs is childcare. Many young children (Europe: 84%, United States: 67%) spend a significant amount of time in childcare [12, 13]. Several studies have examined the role of the childcare setting on the children’s weight status, and the results mostly indicated a higher risk of overweight in children attending childcare [14–17]. This might be due to the influence of the sociocultural environment through the childcare workers’ nutrition and physical activity practices [18] as well as characteristics of the physical environment, such as play materials and playground features [19, 20].

The home is another setting that influences young children’s EBRBs. Parents can influence their children’s behaviours through their general parenting style and specific parenting practices, but also through their influence on the characteristics of the physical home environment [21–23]. Types of environments and settings interact with each other in their influence on behaviour [10, 24, 25]. Given this complex nature of the determinants of EBRBs, a comprehensive, multi-component approach to childhood overweight and obesity prevention is needed [10]. In other words, consistent health-promoting changes across settings should be aimed for [25]. Plus, the different types of environment and the various EBRBs involved in childhood overweight should be taken into account [25].

In general, interventions aimed at the prevention of childhood overweight and obesity focus primarily on one setting. These interventions, targeting either childcare or the home, have shown desired effects on children’s Body Mass Index (BMI) and EBRBs [26–28]. Although previous systematic reviews on childcare interventions took parental involvement into account [27, 29, 30], the evidence is still limited. These reviews used parental involvement in order to explain the effectiveness of childcare interventions however, did not take into account *how* the parents were involved. To our knowledge, only one review specifically studied childcare interventions with parental involvement [31]. This review from 2014, was predominantly explorative, and included only one study in which parents were fully engaged in the intervention [31]. Given the importance of parental involvement in childcare interventions and that it is increasing, an updated and more in-depth study of the literature is needed with a focus on childcare interventions in which the parents are *directly* involved.

There are two types of parental involvement: direct and indirect [32]. Direct parental involvement is defined as “parents’ presence requested at education sessions and/or parents’ attendance and participation requested for family behaviour counselling or parent training sessions” [32]. Indirect parental involvement is defined as “provision of information that did not require parental response, and/or invitations to parents to participate in activities, and/or communications meant to involve parents in intervention activities (e.g. homework assignments)” [32]. Direct parental involvement has been shown to increase intervention effectiveness [32]. Therefore, the current systematic review aims to evaluate the effectiveness of childcare-based interventions with direct parental involvement on weight status and EBRBs of 2–5-year-old children.

## Methods

### Search strategy

A combined search was performed in order to conduct two systematic reviews, one on interventions with parental involvement in the preschool setting (current study) and one in the primary school setting [33]. A list of relevant categories and related search terms and keywords was prepared. The categories of the search were: *intervention participant (*e.g. *child); intervention target behaviours (*e.g. *physical activity/sedentary behaviour or nutrition); school environment (*e.g. *preschool); home environment (*e.g. *parent); intervention;* and *effectiveness studies*. Pubmed, Web of Science, Psycinfo and ERIC were searched. An initial search was performed in June 2016, which was updated in January 2019. Studies published until January 2019 were included in this review. An example of the Pubmed search can be found as supplementary material (Additional file 1: Table S1). Finally, additional studies were found by reference tracking of previous (systematic) reviews and included articles.

### Inclusion and exclusion criteria

Studies were included when they considered a childcare-based intervention targeting physical activity (PA), sedentary behaviour (SB) and/or nutrition behaviour (NB); the target population was children aged 2–5 years old; outcomes measured were BMI, BMI z-score or other weight-related outcomes (e.g. fat percentage, fat free mass) and/or children’s PA (e.g. time spent in total PA or moderate-to-vigorous PA), SB (e.g. screen time or time spent in SB), or NB (e.g. intake of fruits and vegetables, intake of nutrients); and including direct parental involvement [32]. Intervention studies solely describing indirect parental involvement [32] were excluded. Additional exclusion criteria were: not written in English; not applying a pre-post-test design; pilot studies (due to their aim of testing study feasibility instead of effectiveness); interventions in which the preschool was solely used as a location for recruitment and/or venue for the intervention (e.g. afterschool programs or parental education sessions).

### Study selection

After removal of duplicates, the retrieved articles were independently screened by title/abstract by two researchers (IK and SV). Those articles selected for full-text screening were assessed on eligibility independently by IK and SV, taking into account the a priori formulated inclusion and exclusion criteria described above. Discrepancies between selected studies were discussed until consensus was reached. The initial overall agreement between the researchers was 74.5%. In case of no consensus (5 studies), a third researcher (SG) was consulted to determine eligibility.

### Data extraction

Data was extracted on the following study characteristics: design, intervention characteristics (i.e. country, year, setting, duration, follow-up), number of participating childcare centres, participant characteristics (i.e. number of participants, dropout and mean age), and outcomes measured. To understand the interventions better, data was extracted on targeted behaviour, the types of environments involved in the intervention (according to the ANGELO framework [11]), the content and extent of parental involvement, and the effectiveness of the intervention on the evaluated outcomes. To evaluate the effectiveness, data was extracted from the first measurement after intervention (short-term follow-up). Additionally, in case of multiple follow-up measurements, data from the longest follow-up was used as an indication of the long-term effectiveness.

All favourable effects for the intervention group were considered a reflection of effectiveness. Positive effects were determined as: all measures for one outcome (BMI, PA, SB, NB) were significantly favourable for the intervention group. Mixed effects were determined as: at least one of the measures showed significantly favourable results for the intervention group, whereas other measures did not (e.g. significant positive change in motor skill development, but no significant or negative results for PA intensity). Negative effects were determined as: all measures for one outcome significantly favoured the control group. No effects were determined if there were no significant differences between the intervention and control groups.

Where possible, Cohen’s d effect sizes were calculated to indicate the magnitude of effects, either significant or non-significant [34]. If information to calculate the effect size was missing, this information was requested from the authors. A total of eight authors (nine studies) were approached for additional data or clarification of their data. One author replied that he/she no longer had access to the data. Two authors could not be reached at the contact information provided in the article. None of the other authors replied to the request for additional data. The magnitude of the effect size was classified using Lipsey’s cut-off points. An effect size ≤0.32 was considered small, 0.33–0.55 moderate, and ≥ 0.56 large [35]. Data extraction was performed by IK.

### Quality assessment

Methodological quality was assessed using the ‘Effective Public Health Practice Project - Quality assessment tool for Quantitative studies’ that is applicable to quantitative studies of various designs [36]. Two researchers (SV and IK) independently rated the quality of the included studies. The interrater reliability was 72.1%. In case of different ratings, the researchers achieved consensus on the quality score by discussion. The quality of the studies was rated in six categories (selection bias, study design, confounders, blinding, data collection methods, and withdrawal and dropouts). The overall rating was strong when at least four categories were rated as strong and none as weak; moderate when there was one weak rating; and weak in the case of two or more weak ratings [36].

## Results

### Study selection

**Fig. 1:**
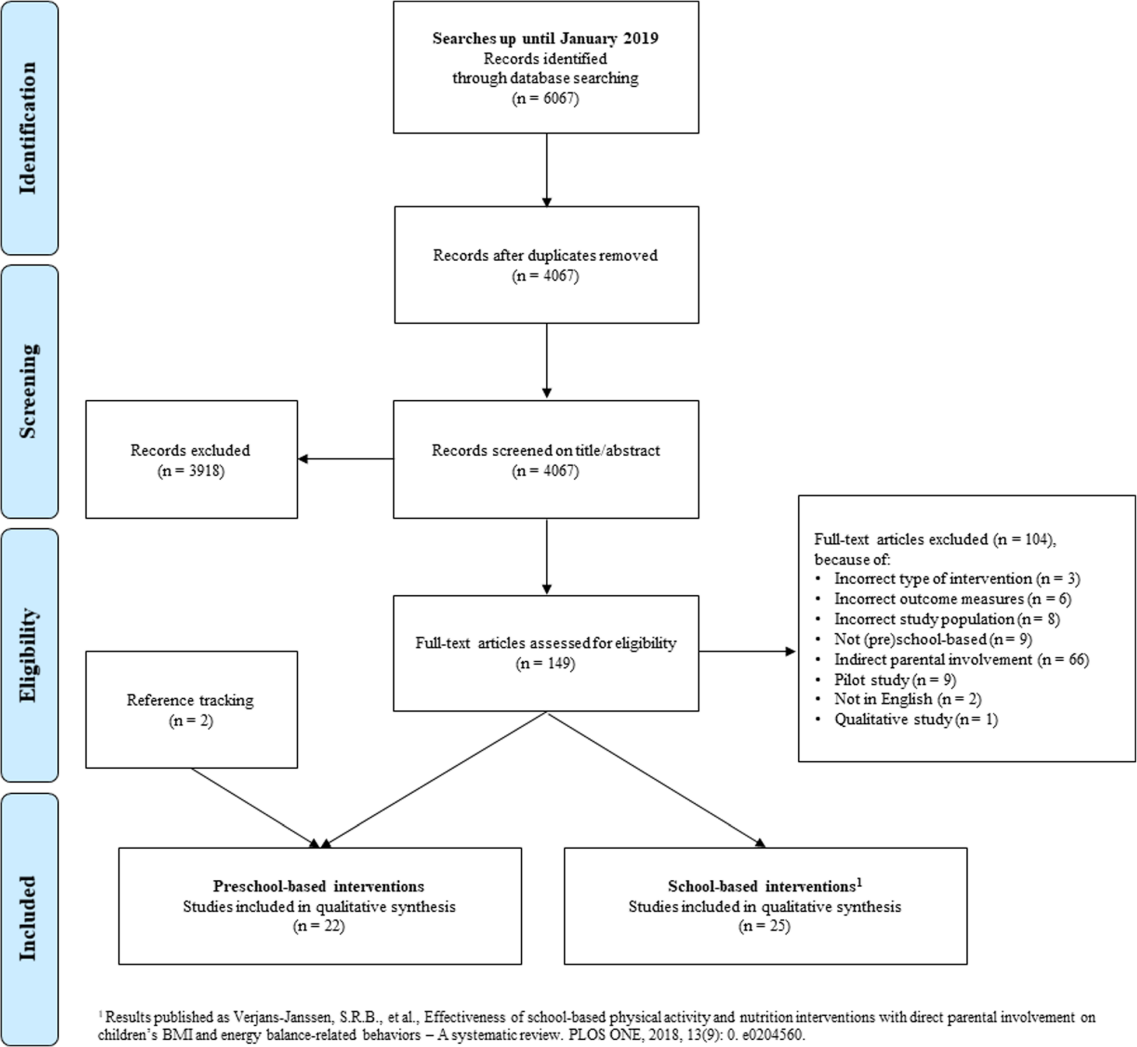
Flowchart of the study selection

The flow diagram of the study selection is shown in Fig. 1. The literature searches resulted in a total of 6067 studies. After removing duplicates, 4067 studies were screened by title/abstract. The full text of 149 records was assessed for eligibility based on the inclusion and exclusion criteria. The most common reason for exclusion was using only indirect parental involvement in the intervention. Other reasons for exclusion were interventions not being (pre-)school-based, pilot studies, and wrong study population. Reference tracking resulted in the inclusion of two additional studies. Eventually, 22 studies on the effectiveness of preschool-based interventions and 25 studies on the effectiveness of primary school-based interventions were included. The results of the primary school-based interventions with direct parental involvement are presented elsewhere [33].

### Study characteristics

The 22 included studies described results from 17 individual interventions. Details on all included studies can be found in Table 1. Nineteen studies adopted a cluster randomized controlled trial (c-RCT) design [37–39, 41–48, 51–55], although three of them described the design as a RCT [42, 55, 56]. Two studies used a quasi-experimental design (no randomization) [49, 50], and one used a retrospective design [40].

**Table 1:** General characteristics of included studies

Study	Study design	Intervention characteristics	No. of participating organisations	Study participant characteristics	Targeted behaviour	Outcome measures and follow-up			
		Country, period, setting, duration		Number, drop-out, mean age		BMI	PA	NB	SB
Adamo et al. (2017)^1^ [51]	Cluster RCT	Canada, Spring 2013 – fall 2014, Childcare centres, 6 months	12 int. childcare centres 6 con. Childcare centres	*N* = 215 34.4% 3.6 ± 0.5	PA	BMI, fat free mass, body fat percentage; 3 and 6 months	Time in total PA, time in MVPA, time in LPA^a^; 3 and 6 months	NA	Time in SB^a^; 3 and 6 months
Cespedes et al. (2013) [37]	Cluster RCT	Colombia, June – October 2009, preschools 5 months	7 int. preschools 7 con. Preschools	*N* = 1216 8.2% NR (range: 3–5 years)	PA, N	BMI^a^; 6 and 18 months	NA	NA	NA
Cruz et al. (2016)^2^ [52]	Cluster RCT	USA, 2008–2010 Head Start Centres, 2 years	8 int. childcare centres 8 con. Childcare centres	*N* = 655 NR† 4.1 ± 0.7 yrs.	PA, N	NA	How often in PA behaviours^b^: - ball playing - dancing - playing active games - jumping - walking; 1 and 2 yrs	NA	NA
Davis et al. (2016)^2^ [56]	Cluster RCT	USA, 2008–2010 Head Start Centres, 2 years	8 int. childcare centres 8 con. Childcare centres	N = 655 NR† 4.1 ± 0.7 yrs	PA, N	BMI^a^; 1 and 2 yrs	NA	NA	NA
De Bock et al. (2013) [38]	Cluster RCT	Germany, 2009–2010 Preschools, 6 months	19 int. preschools 20 con. Preschools	*N* = 809 14.6–31.0% 5.05 ± 0.7 yrs	PA	BMI^a^; 6 and 12 months	Time in MVPA^a^; 6 and 12 months	NA	Time in SB^a^; 6 and 12 months
Gao et al. (2016)^3^ [53]	Cluster RCT	China, 2001–2002, Kindergartens, 10 months	5 int. kindergartens 3 con. Kindergartens	*N* = 2102 16.5% 5.0 ± 0.9 yrs.	N	NA	NA	Breakfast patterns (frequency, food products consumed)^b^; 4 and 10 months	NA
Hu et al. (2010)^3^ [54]	Cluster RCT	China, 2001–2002, Kindergartens, 10 months	5 int. kindergartens 3 con. Kindergartens	N = 2102 16.5% 5.0 ± 0.9	N	BMI^a^; 4 and 10 months	NA	Dietary behaviours^b^; 4 and 10 months	NA
Kaufman-Shriqui et al. (2016) [39]	Cluster RCT	Israel, 2008–2009 Preschools, 3 months	7 int. preschools 4 con. Preschools	*N* = 238 7.6% 5.3 ± 0.54 yrs.	N	BMI^a^ z-score; 3 and 6 months	Time in PA during leisure time^b^; 3 and 6 months	Nutritional habits^b^ (variety of foods consumed, consumption of vegetables, sweets, SSB and water); 3 and 6 months	Daily screentime^b^; 3 and 6 months
Klein et al. (2015) [40]	Retrospectively	Germany, 2006, 2008 Preschools, Unavailable	27 int. preschools 11 cont. Preschools	*N* = 1436 NR 4.7 ± 0.9 yrs	PA, N, SB	BMI (percentile)^a^; 6 months	Motor skill tests^a^ (shuttle run, standing long jump, one leg stand, sit and reach, and lateral jumping); 6 months	NA	NA
Lumeng et al. (2017) [41]	Cluster RCT	USA, 2011–2014 Head Start classrooms, 7 months	4 int. classrooms 2 con. Classrooms	*N* = 697 8.5% 4.11 ± 0.52 yrs	N, SB	BMI z-score^a^; 7 months	Time playing outdoors^b^; 7 months	Intake of servings of specific foods/food groups per day^b^; 7 months	Screentime^b^; 7 months
Natale, Lopez-Mitnik et al. (2014) [42]	(cluster) RCT	USA, NR Childcare centres, 6 months	6 int. childcare centres 2 con. Childcare centres	*N* = 307 NR 3.87 (Range: 2–5 yrs)	PA, N	BMI z-score^a^	Time in moderate PA^b^; 3, 6 and 12 months	Dietary intake at home and childcare^b^; 3, 6 and 12 months	Screentime^b^; 3, 6 and 12 months
Natale, Messiah et al. (2014)^4^ [55]	(cluster) RCT	USA, 2010–2011, Child care centres, 6 months	12 int. childcare centres 20 cont. Childcare centres	*N* = 1211 NR 3.9 ± 0.93 yrs	N, PA, SB	NA	NA	Consumption F/V and junk food^b^; 1 school year	SB^b^; 1 school year
Natale et al. (2017)^4^ [57]	(cluster) RCT	USA, 2010–2011, Child care centres, 6 months	12 int. centres 20 cont. Centres	N = 1211 NR 3.9 ± 0.93 yrs	N, PA, SB	BMI z-scores^a^; 1 school year	NA	Consumption F/V and junk food^b^; 1 school year	NA
Nyberg et al. (2015) [43]^$^	Cluster RCT	Sweden, 2010–2011, Preschools, 6 months	7 int. classrooms 7 cont. Classrooms	*N* = 243 0.9% 6.2 ± 0.3 yrs	N, PA, SB	BMI^a^; 6 and 12 months	Time in PA^a^, PA habits^b^; 6 and 12 months	Intake of indicator foods (F/V, energy-dense products)^b^; 6 and 12 months	SB^b^; 6 and 12 months
Nyberg et al. (2016) [44]^$^	Cluster RCT	Sweden, 2012–2013 Preschools, 6 months	16 int. classrooms 15 cont. Classrooms	*N* = 378 2.6% 6.3 ± 0.3 yrs	N, PA	BMI^a^; 6 and 12 months	Time in PA^a^, PA habits^b^; 6 and 12 months	Intake of indicator foods (F/V, energy-dense products)^b^; 6 and 12 months	SB^b^; 6 and 12 months
Puder et al. (2011) [45]	Cluster RCT	Switzerland, 2008–2009, Preschools, 9 months	20 int. classes 20 cont. Classes	*N* = 727 3,6% 5.2 ± 0.6 yrs	N, PA, SB	BMI^a^, % body fat^a^, Skin fold thickness^a^; 1 school year	Aerobic fitness^a^, motor skills^a^, level of PA^a, b^; 1 school year	Eating habits^b^; 1 school year	Media use^b^; 1 school year
Roth et al. (2015) [46]	Cluster RCT	Germany, 2007–2008, Preschools, 11 months	31 int. preschools 10 cont. Preschools	*N* = 709 14% 4.7 ± 0.6 yrs	PA	BMI^NR^, Skin fold thickness^NR^; 6, 12, and 16 months	Change in MVPA^a^, Composite score of motor skills^a^; 6, 12, and 16 months	NA	NA
Story et al. (2012) [47]	Cluster RCT	USA, 2005–2006, Kindergarten, 11 months	Total of 14 schools, division NR	*N* = 454 NR 5.79 ± 0.51 yrs	PA, N	BMI^a^; 4 rounds (fall kindergarten, spring kindergarten, fall first grade, Spring first grade)	Total PA at school^c^; 4 rounds (fall kindergarten, spring kindergarten, fall first grade, Spring first grade)	% of calories from fat and nutrient content in school meals^a^, food intake at home^b^; 4 rounds (fall kindergarten, spring kindergarten, fall first grade, Spring first grade)	NA
Wasenius et al. (2018)^1^ [58]	Cluster RCT	Canada, Spring 2013 – fall 2014, Childcare centres, 6 months	12 int. childcare centres 6 con. Childcare centres	N = 215 34.4% 3.6 ± 0.5 yrs	PA	BMI^a^; 6 months	Fundamental Motor Skills (FMS)^a^, Total PA^a^; 6 months	NA	NA
Williams et al. (2014) [48]	Cluster RCT	USA, 2010 Child care centres, 6–10 weeks	12 int. childcare centres 12. cont. Childcare centres	*N* = 1143 21.1% 4.4 ± NR yrs	N	NA	NA	At-home consumption of F/V and milk^b^; 1 week post-intervention	NA
Yin et al. (2012) [49]	Quasi experimental	USA, 2010–2011 Child care centres, 8 months	3 int. centres 1 con. Centre	*N* = 384 12% 4.1 ± 0.56 yrs	N, PA	BMI^a^; NR	Gross motor development^a^, Outdoor step count^a^; NR	Dietary intake^a^; NR	NA
Zhou et al. (2014) [50]	Quasi experimental	China, 2010–2011 Child care centres, 12 months	1 int. centre 1 con. Centre	*N* = 387 4.3% 4.40 ± 0.78 yrs.	PA, N	BMI^a^, BMI z-score; 12 months	Physical fitness^a^; 12 months	NA	NA

BMI = Body Mass Index, Con = control, F/V = fruit/vegetables, Int = intervention, LPA = light physical activity; MVPA = moderate-to-vigorous physical activity, N = nutrition, NA = not applicable, NR = not reported, PA = Physical activity, RCT = Randomised Controlled Trial, SB = sedentary behaviour, SSB = sugar sweetened beverages, yrs. = years

†drop-out was not reported at participant level

^1,2,3,4^studies based on the same intervention, but with different outcomes or follow-up. Corresponding numbers indicate the same intervention

^$^Studies used the same intervention, but with different populations

^a^objectively measured

^b^Parent reported

^c^teacher reported

Eight interventions took place in North America [41, 42, 47–49, 51, 52, 55, 57, 58], five in Europe [38, 40, 43–46], two in China [50, 53, 54], one in South America [37] and one in the Middle East [39]. Eight interventions were implemented in childcare centres [41, 42, 48–52, 55], seven in preschools [37–40, 43–46], and two in kindergarten [47, 53, 54].

Most interventions lasted less than one year, ranging from 6 to 10 weeks to 11 months, except for two interventions, one lasting one year [50] and one lasting two years [52, 56]. The interventions took place between 2011 and 2014.

Four interventions targeted NB, PA, and SB [40, 43–45, 55], six interventions targeted NB and PA [37, 42, 47, 49, 50, 52], one intervention targeted NB and SB [41], three interventions targeted only NB [39, 48, 53, 54], and three interventions targeted only PA [38, 46, 51]. All studies, except for four [48, 52, 53, 55], reported on BMI and related outcomes. Fifteen studies reported on a variety of PA-related outcomes [38–47, 49–52, 58], and nine studies reported on SB-related outcomes [38, 39, 41–45, 51, 55]. Thirteen studies reported on NB-related outcomes [39, 41–45, 47–49, 53–55].

### Study quality

Three studies (13.6%) [39, 43, 45] were rated strong for methodological quality (Table 2). Eight studies (36.4%) got a weak rating [41, 42, 47, 51, 52, 56, 57], and the remainder of the studies (50.0%) were rated of moderate quality. Weak or moderate ratings on one of the assessed categories often resulted from a lack of reporting. For example, only two studies reported completely on blinding [45, 51]. Other weak ratings resulted from low recruitment rates [41, 44, 52] or unclear validity and reliability of the measurement instruments [41, 46, 47, 52, 54].

**Table 2:** Quality assessment of the selected studies

Study	Selection bias	Study design	Confounders	Blinding	Data collection methods	Withdrawals and dropouts	Overall rating
Adamo et al. (2017) [51]	Weak	Strong	Weak	Strong	Strong	Weak	Weak
Cespedes et al. (2013) [37]	Strong	Strong	Strong	Moderate	Moderate	Weak	Moderate
Cruz et al. (2016) [52]	Weak	Strong	Strong	Moderate	Weak	Weak	Weak
Davis et al. (2016) [56]	Weak	Strong	Strong	Moderate	Strong	Weak	Weak
De Bock et al. (2013) [38]	Strong	Strong	Strong	Moderate	Strong	Weak	Moderate
Gao et al. (2016) [53]	Moderate	Strong	Weak	Moderate	Strong	Strong	Moderate
Hu et al. (2010) [54]	Strong	Strong	Strong	Moderate	Weak	Strong	Moderate
Kaufman-Shriqui et al. (2016) [39]	Moderate	Strong	Strong	Moderate	Strong	Strong	Strong
Klein et al. (2015) [40]	Moderate	Moderate	Strong	Moderate	Strong	Moderate	Moderate
Lumeng et al. (2017) [41]	Weak	Strong	Strong	Moderate	Weak	Strong	Weak
Natale, Lopez et al. (2014) [42]	Moderate	Strong	Weak	Moderate	Strong	Weak	Weak
Natale, Messiah et al. (2014) [55]	Moderate	Strong	Strong	Moderate	Strong	Weak	Moderate
Natale et al. (2017) [57]	Weak	Strong	Moderate	Moderate	Strong	Weak	Weak
Nyberg et al. (2016) [44]	Weak	Strong	Strong	Moderate	Strong	Strong	Moderate
Nyberg et al. (2015) [43]	Moderate	Strong	Strong	Moderate	Strong	Strong	Strong
Puder et al. (2011) [45]	Strong	Strong	Strong	Strong	Strong	Strong	Strong
Roth et al. (2015) [46]	Moderate	Strong	Strong	Moderate	Weak	Strong	Moderate
Story et al. (2012) [47]	Strong	Strong	Strong	Moderate	Weak	Weak	Weak
Wasenius et al. (2018) [58]	Weak	Strong	Strong	Moderate	Strong	Weak	Weak
Williams et al. (2014) [48]	Moderate	Strong	Strong	Moderate	Strong	Moderate	Moderate
Yin et al. (2012) [49]	Moderate	Strong	Strong	Moderate	Strong	Weak	Moderate
Zhou et al. (2014) [50]	Weak	Strong	Strong	Moderate	Strong	Strong	Moderate

### Intervention components

All interventions consisted of activities to change the sociocultural environment (Table 3). In the preschool component, these were predominantly teacher training sessions or workshops [37, 39, 40, 42, 45–57]. Some interventions added PA lessons or nutrition lessons to the curriculum, to be delivered either by the teachers themselves or by external teachers or experts [38–41, 43–50, 55, 57]. Some interventions provided a manual to support the teacher in the implementation of the intervention [37, 43, 44, 51], while others offered personal assistance [37, 42, 46, 55, 57].

**Table 3:** Intervention components and intervention effectiveness

					Short-term effectiveness				Long-term effectiveness			
Study	Int. comp.	Political env.	Sociocultural env.	Physical env.	BMI	PA	SB	N	BMI	PA	SB	N
Adamo et al. (2017) [51]	Childcare		Two 3-h workshop training sessions for day-care providers, a training manual and weekly schedules. Bimonthly booster sessions during regular hours.	Music developed for PA with a guidebook, starter kit of equipment.	0 ES: 0; 0.39	0 ES: 0	0 ES: 0	NA				
	Parental		Two online training sessions (webinars) or hard copies of training material; ABC Child activities Booklet and bi-weekly postcards.									
Cespedes et al. (2013) [37]	Childcare		Teacher training sessions; personalized working sessions with a research supervisor and a teacher’s guide;	Classroom educational and playful activities (storybooks, posters, videos, games and songs).	0 ES: −0.59	NA	NA	NA	0	NA	NA	NA
	Parental		3 workshops, healthy notes.									
Cruz et al. (2015) [52]	Childcare	Policy changes on food menu	Professional development for teachers through training sessions	New equipment for the classroom; F&V in school menu.	NA	+/0	NA	NA				
	Parental		Take-home materials. Family events.									
	Community			Increase availability and visibility of healthier food options at grocery stores; Provision of recipes and nutrient-related information to families.								
Davis et al. (2016) [56]	Childcare	Policy changes on food menu	Professional development for teachers through training sessions	New equipment for the classroom; F&V in school menu.	0 ES: 0.04	NA	NA	NA				
	Parental		Take-home materials. Family events.									
	Community			Increase availability and visibility of healthier food options at grocery stores; Provision of recipes and nutrient-related information to families.								
De Bock et al. (2013) [38]	Childcare		Twice-weekly 1-h gym class delivered by external gym trainers.	In collaboration with parents 4 various projects were chosen to be implemented at the preschool.	0 ES: 0.01	+/0 ES: 0.06; 0.08	+ ES: −0.06	NA				
	Parent		Parents were actively involved with project list development and selection of the projects to be implemented.									
Gao et al. (2016) [53]	Childcare		Monthly education.	Illustrated book; Series of promotional pictures.	NA	NA	NA	+				
	Parent		Monthly parent-child education (at least 8 lectures or activities); Pamphlets.	Illustrated book.								
Hu et al. (2016) [54]	Childcare		Monthly education.	Illustrated book; Series of promotional pictures.	NR	NA	NA	+/0				
	Parent		Monthly parent-child education (at least 8 lectures or activities); Pamphlets.	Illustrated book.								
Kaufman-Shriqui et al. (2016) [39]	Childcare		Teacher training; Nutritional lessons; PA curriculum (also in control group).		NR	+ ES: 0.18	+ ES:-0.4	+/0 ES: NA	NR	NR	NR	+/0
	Parent		Two meetings for mothers only, one meeting for mothers and children; Weekly newsletter.									
Klein et al. (2015) [40]	Childcare		KIMO&NF: single information session on healthy lifestyle. NF: one physical education class of 60 min per week for 6 months.		+ ES: −0.19; − 0.12	+/−/0 ES: 0.02; 0.37	NA	NA				
	Parent		KIMO&NF: single information session on healthy lifestyle, individual fitness passes with test results.									
Lumeng et al. (2017) [41]	Childcare		POPS: Lessons using children’s stories. POPs+ IYS: Sixty 15–20 min lessons during ‘circle time’ followed by small group activities.		0 ES: −0.12; 0	0 ES: − 0.08; 0.12	0 ES: − 0.17; 0.03	+/0 − 0.32; 0.10				
	Parent		POPS: Eight 75-min weekly lessons with reinforcing telephone contacts. POPS+IYS: 2-h lessons for 12–14 weeks or 10 home visits, homework and follow-up phone calls.									
Natale, Lopez-Mitnik et al. (2014) [42]	Childcare	Development of nutrition and PA policies	Two trainings for teachers and staff; Weekly technical assistance visit.	Modifying menus to fit the new policies.	0 ES:-0.04	0 ES: NA	+	+				
	Parent		Monthly educational dinner.	Receiving healthy snack bag after completion of at-home activities.								
Natale, Messiah et al. (2014) [55]	Childcare	Drink policy Snack policy Physical activity policy Screen time policy	Six monthly trainings; Child curriculum; Weekly technical assistance for child curriculum.	Food tastings Music and movement CDs, rainy day activities and equipment.	NA	NA	+	+				
	Parent		Six monthly trainings.									
Natale et al. (2017) [57]	Childcare	Drink policy Snack policy Physical activity policy Screen time policy	Six monthly trainings; Child curriculum; Weekly technical assistance for child curriculum.	Food tastings Music and movement CDs, rainy day activities and equipment.	+	NA	NA	0				
	Parent		Six monthly trainings.									
Nyberg et al. (2015) [43]	Childcare		Ten 30-min teacher-led sessions with teacher manual and workbook	Tool-box and extra educational materials.	0 ES: −0.04	0 ES: −0.33; − 0.12	0 ES: 0.06; 0.07	0 ES: − 0.88; 0.40	0	0 ES: − 0.18; 0.07	0 ES:-0.14; − 0.13	0 ES: −1.35; 0.61
	Parent		Brochure; Two motivational interviewing sessions; Homework assignments for the children.									
Nyberg et al. (2016) [44]	Childcare		Ten 30-min teacher-led sessions with teacher manual and workbook.	Tool-box and extra educational materials.	0 ES: −0.02	0 ES: − 0.16; − 0.06	0 ES: − 0.03; 0.03	+/0 ES: − 1.06; 0.20;	0 ES: 0.01	0 ES − 0.18; − 0.15	+/0 ES: − 0.22; − 0.21	0 ES: − 0.82; 0.03
	Parent		Brochure; One group meeting at school to discuss the brochure; Two individual sessions of MI; Homework assignments for the children.									
Puder et al. (2011) [45]	Childcare		Two teacher workshops; PA lessons 4 times per week (first by HP and taken over by PT); Weekly nutrition lessons; extracurricular PA activities.	Additional sports equipment for the PA lessons; Infrastructural changes in the building.	+/0 ES: −0.23; 0.07	+/0 ES: − 0.13; 0.22	+	+				
	Parent		PA and nutrition card that the child took home; morning event; three information evenings; information booklet.	CD with music for the PA cards.								
Roth et al. (2015) [46]	Childcare		Daily 30-min PA lessons provided by PT; Two afternoon workshops; supervision visits; Cards with educational content to help teachers plan and realise PA lessons.		0 ES: −0.06; 0.023	+/0 ES: − 0.13; 0.20	NA	NA	0 ES: 0.03; 0.05	+/0 ES: −0.05; 0.23	NA	NA
	Parent		Three educational seminars; Booklet on healthy eating, PA; booklets and letter on the content of the seminars	Homework cards with activity games and motor tasks.								
Story et al. (2012) [47]	Childcare		School PE, class walks outdoors, in-class action breaks, and active recess; Training of PE teachers; Training of school food-service staff; teacher training.	‘Action toolbox’; playground equipment; non-food rewards for classroom performance.	+/0 ES: −0.24; 0.07	0 ES: NA	NA	+/0 ES:-2.22; 1.40				
	Parent		Three family night events; motivational encouragement telephone calls; quarterly newsletter.	Take-home incentives related to PA or nutrition.								
Wasenius et al. (2018) [58]	Childcare		Two 3-h workshop training sessions for day-care providers, a training manual and weekly schedules. Bimonthly booster sessions during regular hours.	Music developed for PA with a guidebook, starter kit of equipment.	NR	+/0 ES: 0.53; 1.49	NA	NA				
	Parent		Two online training sessions (webinars) or hard copies of training material; ABC Child activities Booklet and bi-weekly postcards.									
Williams et al. (2014) [48]	Childcare	Policy improvement to enhance nutrition.	Two classes for staff; 30 min lessons for children (selected six out of ten possible modules).		NA	NA	NA	+/0 ES: 0; 0.18				
	Parent		30–60 min parent classes (the same selected six out of ten possible modules).	take-home materials and activities.								
Yin et al. (2012) [49]	Childcare		Teacher training to implement a gross motor skills program during daily outdoor play; provision of structured play activities the first 15–20 min of outdoor play; Sesame Street Workshop Healthy Habits for Life (HHL, nine modules); food-tasting activities and contests; 6-h initial training of staff with follow-up trainings.	Activity cards and equipment for the motor skills program; children’s storybooks with nutrition and PA themes	0 ES: − 0.04	+ ES: 0.03	NA	+/0 ES: NA				
	Parent		Eight newsletters about HHL; parent delivered poster sessions at dismissal time; information scavenger hunt.	Take-home bag with a storybook, family activities and an interactive game; healthy snack for the child after viewing the posters.								
Zhou et al. (2014) [50]	Childcare	Policy related to outdoor play time and physical education.	Bi-weekly 60-min training sessions (to 20 h); physical education curriculum for outdoor play period; two training sessions (3 h) for food services workers.	Portable play equipment; poster of children playing on the outside walls; game markings on the outdoor playground and indoor play space; permanent markings for skipping and hopping both indoors and outdoors.	0 ES: − 0.61; 0.32	+ ES: − 0.55; 0.45	NA	NA				
	Parent		Monthly health education seminars; 12 monthly newsletters; interactive website; family events for both parent and child.									
	Community		Training of neighbourhood associations staff; neighbourhood events; hosting sports day for families.	Renovation of neighbourhood playgrounds; installation of child’s play equipment								

Int. = intervention; BMI = Body Mass Index; HS + POPS = Head Start + Preschool Obesity Prevention Series; IYS = Incredible Years Series; KiMo = Kindergarten Mobile; N = Nutrition; NA = Not Applicable; NF-P = Nursery Fit-Participated; NF-NP=Nursery Fit-Not Participated; NR = Not Reported; SB = Sedentary Behaviour; PA = Physical Activity

*Effectiveness is presented as positive effects (+), all effects significantly favoured the intervention group; mixed effects (+/0/−), one of the effects significantly favoured the intervention group, the other effects were not significant or favoured the control group; negative effects (−), all effects significantly favoured the control group

**Effect sizes are only provided for studies and outcomes for which effect sizes could be calculated. The positive or negative indicator shows the direction of effect. Depending on the outcome, this favoured the intervention group or the control group

In the family component, intervention activities to change the sociocultural environment were mostly parent training sessions or workshops [37, 39–42, 45, 46, 48–51, 53–55, 57]. Some interventions organized family events [45, 47, 49, 50, 52, 56]. One intervention took a participatory approach and actively involved parents in the selection of projects to be implemented that would affect both the preschool and family component [38]. In addition to these direct parental involvement activities, almost all interventions also used indirect parental involvement activities such as newsletters, information leaflets, and homework assignments [37, 39–41, 43–47, 49–54, 56].

Fourteen interventions included activities to change the physical environment in the preschool [37, 38, 42–47, 49–57]. The most commonly used intervention activities were providing equipment for PA [37, 47, 50–52, 55–57] and intervention-specific materials [43–46, 49]. Other activities were food menu changes [42, 52, 56], providing children’s storybooks related to nutrition or PA [49, 53, 54], and permanent markings on indoor and outdoor play areas [50]. In the family component, seven interventions implemented activities in the physical environment [42, 45–49, 53, 54]. These included take-home materials and activities [45, 46, 48, 49, 53, 54] and take-home healthy nutrition or PA-related incentives [42, 47].

Five interventions tried to change the political environment in the preschool component [42, 48, 50, 52, 55–57] by formulating or changing policies related to NB [48, 52, 56], PA [50], or both [42, 55, 57]. None of the interventions included activities to change the economic environment.

In addition to the preschool and family components, two interventions also included a community component [50, 52, 56]. For example, neighbourhood events were organized [50], or healthy food options were made increasingly available and visible in grocery stores [52, 56]. One intervention aimed at changes in the sociocultural environment through training of neighbourhood association staff, neighbourhood events, and a sports day for families [50]. Both interventions included activities to change the physical environment through increasing the availability and visibility of healthy food options at grocery stores [52, 56] and renovation of neighbourhood playgrounds and installing children’s play equipment [50].

### Study effects

#### Effects on weight-related outcomes

Of the eighteen studies reporting on weight-related outcomes, eleven (61.1%) found favourable results for the intervention group for one of the weight-related outcomes [37, 40–45, 47, 49, 50, 57] (Table 4). Of these eleven studies, two were positively effective on all weight-related outcomes [40, 57], and two found mixed results [45, 47]. The other studies did not find significant differences between the study groups, and some also found unfavourable results regarding some of their weight-related outcomes [47, 50]. Effect sizes were calculated for all studies except one [57]. All effects on weight-related outcomes were small, except for Cespedes et al. (2013), who found a moderate favourable effect size [37]. Four studies found unfavourable effects for the intervention group [38, 46, 51, 56]. The results of these four studies were all non-significant, with small effect sizes, except for Adamo et al. (2017), who found a moderate effect size for body fat percentage [51].

**Table 4:** Intervention effectiveness based on reported results with effect sizes where available

Study	BMI/BMI z-score	Physical activity	Sedentary behaviour	Nutrition behaviour
Adamo et al. (2017) [51]	Short term follow-up: No change in BMI in the intervention group (0.0 kg/m^2^) compared to a decrease in the control group (− 0.5 kg/m^2^) (*p* = 0.155) ES 0.24 Larger increase of fat mass in the intervention group (0.6 kg) compared to the control group (0.2 kg) (*p* = 0.234) ES 0.30 Increase in fat-free mass in both the intervention (0.7 kg) and the control group (0.7 kg) (*p* = 0.876) ES 0 Increase in fat percent in the intervention group (1.7%) compared to a decrease in the control group (− 0.6%) (*p* = 0.253) ES 0.39	Short term follow-up: Increase in total physical activity in both the intervention group (1.6 min/h) and the control group (1.6 min/h) (*p* = 0.995) ES 0 Increase in MVPA in both the intervention group (1.3 min/h) and the control group (1.3 min/h) (*p* = 0.932) ES 0 Increase in LPA in both the intervention (0.3 min/h) and control group (0.3 min/h) (*p* = 0.955) ES 0	Short term follow-up: Decrease in sedentary time in both the intervention (− 1.6 min/h) and the control group (− 1.6 min/h) (p = 0.995) ES 0	NA
Cespedes et al. (2013) [37]	Short term follow-up: Smaller increase in BMI in the intervention (0.58 kg/m^2^) compared to the control group (0.63 kg/m^2^) (*p* = 0.193) ES − 0.59 Long term measurement: No significant differences between the intervention and control group (*p* = 0.5, no data provided).	NA	NA	NA
Cruz et al. (2016) [52]	NA	Short term follow-up: Increase in proportion ‘often’ ball playing in intervention group (+ 8.2%) compared to a decrease in the control group (− 4.5%) (ns) Increase in proportion ‘often’ dancing in intervention group (+ 16.1%) compared to a decrease in the control group (− 10.6%) (*p* < 0.01) Larger increase in proportion ‘often’ playing active games in intervention group (+ 10.8%) compared to the control group (+ 5.9%) (ns) Larger increase in proportion ‘often’ jumping in intervention group (+ 11.8%) compared to the control group (+ 5.4%) (ns) Increase in proportion ‘often’ walking in intervention group (+ 2.5%) compared to a decrease in the control group (− 1.3%) (ns)	NA	NA
Davis et al. (2016) [56]	Short term follow-up: Larger increase in BMI z-score in the intervention group (0.17) compared to the control group (0.11) (*p* = 0.34) ES 0.036	NA	NA	NA
De Bock et al. (2013) [38]	Short term follow-up: No differences in mean change in BMI (0.064 kg/m^2^) between intervention and control group (*p* = 0.41) ES 0.01 No differences in mean change in body fat (0.21%) between intervention and control group (*p* = 0.32)	Short term follow-up: Increase of mean counts per 15-s interval (+ 1.38) in intervention group compared to control group (*p* = 0.019) ES 0.08 No difference in MVPA (+ 0.97 min) between intervention and control group (*p* > 0.1) ES 0.06	Short term follow-up: Decrease in time in sedentary behaviour (− 11 min) in the intervention group compared to control group (*p* = 0.014) ES − 0.06	NA
Gao et al. (2016) [53]	NA	NA	NA	Short term follow-up: Increase in daily breakfast frequency in the intervention group (+ 1.1%) compared to a decrease in the control group (− 1.9) (*p* = 0.02) Increase in quantity of food for breakfast in the intervention group compared to a decrease in the control group (*p* < 0.001) More high-in-nutrient food types in breakfast in the intervention group compared to more high-in-energy food types in the control group (p < 0.001)
Hu et al. (2010) [54]	NR	NA	NA	Short term follow-up: Some unhealthy diet-related behaviours were significantly different between the intervention and control groups (*p* < 0.05), while others showed no significant difference. Improvement in healthy diet-related behaviours in the intervention group (p < 0.05).
Kaufman-Shriqui et al. (2016) [39]	Follow-up not indicated: Reduction of BMI z-score (− 0.1) in total study population (*p* = 0.003). No group-specific scores reported.	Follow-up not indicated: Decrease of mean PA time in control group (− 0.42 h) compared to intervention group (− 0.21 h, *p* = 0.03) ES 0.18	Follow-up not indicated: Increase of screen time in control group (+ 0.54 h) compared to no change in intervention group (*p* = 0.001) ES − 0.4	Short term follow-up: Greater increase in food variety (intervention + 26.5%, control + 7.6%); daily vegetable consumption (intervention + 24.7%, control + 9.2%), and habitual water drinking (intervention + 21.3%, control + 10.8%) in the intervention group compared to the control group, all *p* < 0.05. Greater decrease in daily consumption of SSB in the intervention group (− 19.2%) compared to the control group (− 13.6%, p = 0.02). Non-significant smaller decrease in daily consumption of sweet and candies in the intervention group (− 17.7%) compared to the control group (− 18.2%, *p* = 0.08) Long term follow-up: Greater increases in food variety (intervention + 25.3%, control + 8.1%), daily vegetable consumption (Intervention + 22.3%, control + 8.8%), and habitual water drinking (intervention + 19%, control + 11.9%) in intervention group compared to control group (all p < 0.05). Decrease in daily consumption of SSB in the intervention group (− 15.3%) compared to control group (− 8.3%) (*p* = 0.05) No significant difference between intervention group (− 22.9%) and control group (− 15.2%) in consumption of sweet and candies on daily basis (*p* = 0.13).
Klein et al. (2015) [40]	Short term follow-up: Significant decrease in BMI in group KiMo (− 0.1 kg/m^2^), NF-P (− 0.1 kg/m^2^) and NF-NP (− 0.2 kg/m^2^) compared to an increase in control group (all p < 0.001) ES − 0.13, − 0.12, − 0.19, respectively	Short term follow-up: Motor tests: Non-significant differences in Shuttle Run between groups (KiMo − 1.1 s, NF-P − 0.8 s, NF-NP − 1.0 s and CG − 1.3 s) ES 0.06, 0.17, 0.1, respectively Non-significant differences in Standing Long Jump between groups (KiMo + 12.6 cm, NF-P + 10.8 cm, NF-NP + 13.1 cm, CG + 8.8 cm) ES 0.15, 0.08, 0.17, respectively Significant differences in Sit and Reach between KiMo (+ 0.7 cm, p < 0.001), NF-P (+ 0.3, *p* = 0.007), NF-NP (+ 0.6 cm, p < 0.001) and control group (− 0.6 cm) ES 0.27, 0.20, 0.27, respectively Significant negative difference in One Leg Stand between KiMo (− 2.0 ground contacts, p < 0.001), NF-P (− 2.8 ground contacts, *p* = 0.035) and control group (− 3.2 ground contacts) ES 0.16, 0.05, respectively Non-significant difference between NF-NP (− 3.2 ground contacts) and control group (− 3.2 ground contacts) ES 0 Non-significant differences in Lateral Jumping between KiMo (+ 4.4 jumps), NF-P (+ 4.7 jumps), NF-NP (+ 4.8 jumps), and control group (+ 4.2 jumps) ES 0.02, 0.05, 0.06, respectively	NA	NA
Lumeng et al. (2017) [41]	Short term follow-up: Non-significant difference in percentage overweight or obese between HS + POPS (− 2.3%, *p* = 0.35), HS + POPS+IYS (− 0.6%, *p* = 0.77) and HS (+ 0.6%) Non-significant differences in percentage obese between HS + POPS (− 2.9%, *p* = 0.16), HS + POPS+IYS (− 2.1%, *p* = 0.33) and HS (+ 0.8%) Non-significant differences in BMI z-score in children overweight or obese at baseline between HS + POPS (− 0.11, *p* = 0.98), HS + POPS+IYS (− 0.16, *p* = 0.44) and HS (− 0.11) ES 0, − 0.12, respectively	Short term follow-up: Non-significant differences in outdoor play between HS + POPS (− 0.82 h/d, *p* = 0.48), HS + POPS+IYS (− 0.47 h/d, *p* = 0.25) and HS (− 0.68 h/d) ES − 0.08, 0.12, respectively	Short term follow-up: Non-significant difference in screen time between HS + POPS (+ 0.55 h/d, *p* = 0.75), HS + POPS+IYS (+ 0.24 h/d, *p* = 0.11) and HS (+ 0.5 h/d) ES 0.03, − 0.17, respectively	Short term follow-up: Non-significant differences in vegetable servings/day between HS + POPS (− 0.02, *p* = 0.90), HS + POPS+IYS (− 0.05, *p* = 0.88) and HS (− 0.03) ES 0.01, − 0.02, respectively Non-significant differences in whole fruit servings/day between HS + POPS (+ 0.05, p 0.86), HS + POPS+IYS (− 0.02, *p* = 0.60) and HS (+ 0.03) ES 0.02, − 0.04, respectively Non-significant differences in fruit juice servings/day between HS + POPS (− 0.21, p = 0.77), HS + POPS+IYS (− 0.06, *p* = 0.39) and HS (− 0.17) ES − 0.03, 0.10, respectively Non-significant difference in SSB servings/day between HS + POPS (+ 0.01, *p* = 0.12) and HS (+ 0.14) ES − 0.20 Significant difference in SSB servings/day between HS + POPS+IYS (− 0.07, *p* = 0.005) and HS (+ 0.14) ES − 0.32
Natale, Lopez-Mitnik et al. (2014) [42]	Short term follow-up: Less increase in BMI z-score in the intervention group (+ 0.05) compared to the control group (+ 0.16) (NS) ES − 0.04	Short term follow-up: No significant differences between intervention and control group (no data reported).	Follow-up not indicated: Significantly more time spent on the computer (p < 0.01) and watching TV (*p* < 0.0001) in the control group compared to the intervention group at school (no data reported).	Follow-up not indicated: During school time: Intervention group decreased mean junk food consumption, while the control group increased consumption. Intervention group increased mean fresh fruit and vegetable consumption. Intervention groups decreased juice consumption. Intervention group increased 1% milk consumption. Control group decreased water consumption. For all outcomes no data were reported.
Natale, Messiah et al. (2014) [55]	NA	NA	Short term follow-up: The intervention group decreased sedentary behaviour, compared to an increase in the control group (*p* < 0.004).	Short term follow-up: No change in fruit/vegetable consumption in the intervention group, compared to a decrease in the control group (*p* < 0.05). The intervention group decreased the consumption of junk food, compared to an increase in the control group (*p* = 0.01).
Natale et al. (2017) [57]	Short term follow-up: The intervention group had a negative slope (β = − 1.95, *p* = 0.04) in BMI percentile growth curve, indicating a significant positive change in PBMI over time.	NA	NA	Short term follow-up: No significant difference between groups in change over time in children’s fruit/vegetable consumption (β = 0.04, *p* = 0.34) and children’s unhealthy food consumption (β = 0.01, *p* = 0.80).
Nyberg et al. (2015) [43]	Short term follow-up: No significant difference in BMIsds between intervention (∆-0.11) and control group (∆-0.06) ES − 0.04. No significant difference in change of prevalence of underweight (∆ = 1.6, *p* = 0.53), normal weight (∆ = − 1.9, *p* = 0.65), overweight (∆ = 2.3, *p* = 0.54), obese (∆ = − 1.8, p = 0.16). Long term follow-up: No significant difference in change of prevalence of underweight (∆-0.8, *p* = 0.69), normal weight (∆ + 0.9, *p* = 0.61), overweight (∆ + 4.7, *p* = 0.43), and obesity (∆-1.8, *p* = 0.37) between the intervention and control group. Outcomes on BMIsds not reported.	Short term follow-up: No significant differences between the intervention and control group in TPA (cpm, β = − 21.2, *p* = 0.58) or MVPA (minutes, β = − 4.9, p = 0.33) ES − 0.12, − 0.13 resp. Non-significant difference in ‘child taken to activity in the last week’ (time/week) between intervention and control group (β = − 0.48, *p* = 0.07) ES − 0.33 Long term follow-up: No significant differences between the intervention group and control group in TPA (cpm, β = − 15.0, *p* = 0.51) or MVPA (minutes, β = + 2.7, p = 0.60) ES − 0.09, 0.07 resp. No significant difference in ‘child taken to activity in the last week’ (time/week) between intervention and control group (β = − 0.27, *p* = 0.22) ES − 0.18	Short term follow-up: No significant difference in % time spent sedentary (β = 0.4, *p* = 0.59) between the intervention and the control group ES 0.07. No significant difference between the intervention and the control group in screen time viewing (min/day, β = − 3.59, *p* = 0.76) ES − 0.06. Long term follow-up: No significant differences in % time spent sedentary (β = − 0.8, *p* = 0.27) between the intervention and control group. ES − 0.13 No significant difference in screen time viewing (min/day) between intervention and control group (β = − 8.23, *p* = 0.29) ES − 0.14	Short term follow-up: No significant differences of ‘servings in the precious weekday’ between intervention and control group for fruit juice (β = − 0.20, *p* = 0.38) ES − 0.25; soft drink/sugar syrup (β = − 0.37, *p* = 0.23) ES − 0.88; milk (β = 0.04, *p* = 0.71) ES 0.04; flavoured milk (β = 0.04, *p* = 0.92) ES 0.09; vegetables (β = 0.09, *p* = 0.44) ES 0.08; snacks (β = − 0.28, p = 0.44) ES − 0.48; fruit (β = 0.11, *p* = 0.26) ES 0.08; sweets (β = − 0.003, *p* = 0.99) ES − 0.004; cakes/buns/cookies (β = − 0.25, *p* = 0.24) ES − 0.30; ice-cream (β = 0.08, p = 0.69) ES 0.09. Significant difference between the intervention and the control group for ‘usual servings of vegetables per day’ (β = 0.26, *p* = 0.003) ES 0.40 Long term follow-up: No significant difference of ‘servings in the previous weekday, between intervention or control group for fruit juice (β = − 0.21, *p* = 0.41) ES − 0.26; soft drink/sugar syrup (β = + 0.20, *p* = 0.63) ES 0.45; milk (β = − 0.01, *p* = 0.95) ES − 0.01; flavoured milk (β = − 0.18, *p* = 0.67) ES − 0.43; vegetables (β = + 0.05, p = 0.67) ES 0.05; snacks (β = − 0.67, *p* = 0.30) ES − 1.35; fruit (β = + 0.13, p = 0.23) ES 0.10; sweets (β = + 0.49, p = 0.23) ES 0.61; cakes/buns/cookies (β = + 0.38, p = 0.24) ES 0.47; ice-cream (β = + 0.41, *p* = 0.18) ES 0.46. No significant difference in usual servings of vegetables per day between the intervention and control group (β = + 0.14, *p* = 0.14) ES 0.21
Nyberg et al. (2016) [44]	Short term follow-up: No significant differences in BMI sds scores between intervention and control group (β = − 0.03, *p* = 0.46) ES − 0.02 Long term follow-up: No significant differences in BMI sds scores between the intervention and control group (β = 0.013, *p* = 0.79) ES 0.01	Short term follow-up: No significant differences between the intervention and the control group for TPA (cpm, β = − 30.1, p = 0.18) or MVPA (minutes, β = − 1.5, *p* = 0.55) ES − 0.16, − 0.06 resp. Long term follow-up: No significant differences between the intervention group and control group in TPA (cpm, β = − 34.8, *p* = 0.13) or MVPA (minutes, β = − 3.6, *p* = 0.19) ES − 0.18, − 0.15 resp.	Short term follow-up: No significant difference in sedentary time in minutes between intervention and control group (β = 1.5, *p* = 0.68) ES 0.03 No significant difference in screen time (min/day) between the intervention and the control group (β = − 2.6, p = 0.79) ES − 0.03 Long term follow-up: A significant difference on sedentary time in minutes (β = − 9.2, *p* = 0.03) between the intervention and control group ES − 0.21. No significant difference in screen time (min/day) between the intervention and the control group (β = − 16.5, *p* = 0.10) ES − 0.22.	Short term follow-up: No significant differences of ‘servings in the previous weekday’ between intervention and control group for fruit juice (β = − 0.24, *p* = 0.16) ES − 0.37; soft drink/sugar syrup (β = − 0.28, *p* = 0.25) ES − 0.60; flavoured milk (β = − 0.47, *p* = 0.15) ES − 0.93; vegetables (β = 0.15, p = 0.22) ES 0.20; snacks (β = − 0.57, *p* = 0.08) ES − 1.06; fruits (β = − 0.15, p = 0.13) ES − 0.16; sweets/chocolate (β = − 0.38, p = 0.10) ES − 0.58; cakes/buns/cookies (β = 0.00, *p* = 1.00) ES 0; ice cream (β-0.22, p = 0.22) ES − 0.29 Significant difference on aggregated variables ‘unhealthy food’ (β = − 0.32, *p* = 0.01); ‘unhealthy drink’ (β = − 0.51, p = 0.01) between intervention and control group. No significant difference in aggregated variable ‘healthy food’ (β = − 0.02, p = 0.79) between the intervention and control group. Long term follow-up: No significant differences of ‘servings in the previous weekday’ between intervention and control group for fruit juice (β = − 0.09, *p* = 0.70) ES − 0.14; soft drink/sugar syrup (β = + 0.02, p = 0.95) 0.04; flavoured milk (β = − 0.04, p = 0.92) ES − 0.07; vegetables (β = + 0.02, *p* = 0.85) ES 0.03; snacks (β = − 0.46, p = 0.19) ES − 0.82; fruits (β = + 0.03, *p* = 0.76) ES 0.03; sweets/chocolate (β = − 0.26, p = 0.29) ES − 0.39; cakes/buns/cookies (β = − 0.33, *p* = 0.12) ES − 0.43; ice-cream (β = − 0.22, p = 0.30) ES − 0.29. No significant differences on aggregated variables ‘unhealthy food’ (β = − 0.15, *p* = 0.42); ‘unhealthy drink’ (β = 0.05, *p* = 0.83); and ‘healthy food’ (β = − 0.03, p = 0.68) between the intervention and the control group.
Puder et al. (2011) [45]	Short term follow-up: No significant difference in BMI change between the intervention and control group (∆-0.07, *p* = 0.31). ES 0.07 Significant reductions in percentage body fat (∆-1.1, *p* = 0.02) and sum of skinfolds (∆-2.78, *p* = 0.001) in the intervention group compared to the control group. ES − 0.15, − 0.02, respectively Significantly lower increase in waist circumference (∆-1.0, p = 0.001) in the intervention group compared to the control group. ES − 0.24	Short term follow-up: Significantly higher increase in aerobic fitness in the intervention group compared to the control group (∆ + 0.32, p = 0.01). ES 0.22 Significant improvement in motor agility (time to perform an obstacle course) in the intervention group compared to the control group (∆-0.54, *p* = 0.004). ES − 0.13 No significant difference in dynamic balance (∆ + 0.2, *p* = 0.35) and static balance (∆ = + 19.4, p = 0.18) between the intervention and control group. ES 0.06, 0.04, respectively No significant difference in TPA (cpm, ∆-12.3, *p* = 0.54) between the intervention and control group. ES 0.012	Short term follow-up: Significant difference in media use (min/day) between the intervention and control group (∆-13.4, p = 0.03). ES − 0.22	Short term follow-up: Significant difference in proportion healthy eaters between the intervention and the control group (∆ + 1.9, *p* = 0.04).
Roth et al. (2015) [46]	Short term follow-up: No significant difference between the intervention and control group on BMI (centile, ∆ + 0.244, *p* = 0.857); and sum of four skinfolds (mm, ∆ + 1.548, *p* = 0.272). ES 0.023, − 0.06 respectively Long term follow-up: No significant difference between the intervention and the control group on BMI (centile, ∆ + 0.103, *p* = 0.949); and sum of four skinfolds (mm, ∆ + 0.305, *p* = 0.846). ES 0.05, 0.03, respectively	Short term follow-up: No significant (Bonferroni adjusted α) difference in MVPA between the intervention and the control group (∆ + 0.005, *p* = 0.049). Significant increase in motor skills performance (z-score) in children in the intervention group compared to the control group (∆ + 0.623, p = 0.001). Significant improvements in explosive leg strength (cm, ∆ + 3.209, p = 0.004) ES − 0.07; jumping coordination (jumps, ∆ + 1.451, *p* = 0.019) ES 0.20; and static balance (tips, ∆-1.474, *p* = 0.032) ES − 0.13, in the intervention group compared to the control group. No significant improvements in agility (seconds, ∆-0.628, *p* = 0.060) ES − 0.09; dynamic balance (% failure, ∆-0.015, *p* = 0.617); and throwing ability (% failure, ∆-0.020, *p* = 0.465). Long term follow-up No significant difference in MVPA between the intervention and the control group (∆ + 0.006, *p* = 0.859). Significant increase in motor skills performance (z-score) in children in the intervention group compared to the control group (∆ = + 0.590, *p* = 0.007). Significantly better improvements in the intervention group in agility (seconds, ∆-0.689, *p* = 0.034) ES − 0.11 and explosive leg strength (cm, ∆ = + 4.041, p = 0.007) ES 0.23. No significant differences between the intervention group and control group in static balance (tips, ∆-0.306, *p* = 0.629) ES − 0.05; jumping coordination (jumps, ∆ + 1.276, *p* = 0.089) ES 0.18; dynamic balance (% failure, ∆ + 0.051, *p* = 0.220); and throwing ability (% failure, ∆ + 0.006, *p* = 0898).	NA	NA
Story et al. (2012) [47]	Short term follow-up: No significant difference between the intervention and the control group in BMI (kg/m^2^, ∆ + 0.34, *p* = 0.057) ES 0.07; BMI z (∆ + 0.01, *p* = 0.904) ES 0; triceps (mm, ∆ + 0.02, *p* = 0.978) ES 0.003; subscapular (mm, ∆ + 0.05, *p* = 0.909) ES 0.005; % body fat (∆0.90, *p* = 0.122) ES 0.07; and % obese (∆ + 2.11, *p* = 0.503) ES 0.04. A significant difference in % overweight (∆-10.14, p = 0.019) between the intervention and the control group. ES − 0.24	Short term follow-up: A greater mean in PA (combined from recess and PE class in min/week) in the intervention group compared to the control group (NS).	NA	Short term follow-up: Nutrients from school menus: A significant difference between the intervention and control group in % total fat calories (∆-8.00, p = 0.004); and % calories saturated fat (∆-4.08, *p* = 0.002). No significant difference between the intervention and control group in kilocalories (∆-37.3, *p* = 0.691) ES − 0.0007; carbohydrate (g, ∆ + 11.5, *p* = 0.487) ES 1.4; protein (g, ∆-0.26, *p* = 0.933) ES − 0.13; fat (g, ∆-7.81, *p* = 0.085) ES − 2.22; iron (mg, − 0.16, *p* = 0.877) ES − 0.33; magnesium (mg, ∆ + 3.9, *p* = 0.740) ES − 0.79; calcium (mg, ∆ + 64, *p* = 0.827) ES 0.39; sodium (mg, ∆-96, *p* = 0.624) ES − 0.84; vitamin A (RAE, ∆ = + 36.6, *p* = 0.643) ES 1.01; vitamin D IU (∆ = + 0.28, *p* = 0.505) ES 1.33; folate (mg, ∆ = + 13.6, *p* = 0.581) ES 1.01; and sugar added (g, ∆-2.66, *p* = 0.763) ES − 0.36 Food intake reported by parents: Significant difference in intake times per day of sweetened beverages (∆-0.28, *p* = 0.024); whole milk (∆-0.22, *p* = 0.011); and chocolate milk (∆-0.17, *p* = 0.025) between the intervention and control group. No significant difference in intake times per day of vegetables (∆ + 0.02, *p* = 0.788); fruits (∆ + 0.07, *p* = 0.269); skim milk (∆ + 0.12, *p* = 0.138); 100% juice (∆-0.03, *p* = 0.689); bottled water (∆ + 0.09, *p* = 0.413); and fast food (∆ + 0.04, *p* = 0.374.
Wasenius et al. (2018) [58]	NR	Short term follow-up: Significant difference in locomotor skills between intervention and control group (∆ + 2.4, *p* < 0.001) ES 1.31. No significant difference between intervention and control group on object control skills (∆ + 0.5, *p* = 1.0) ES 0.53, sum of raw scores (∆ + 2.8, *p* = 0.333) ES 1.48 or Gross Motor Quotient (∆ + 3.2, *p* = 0.498) ES 1.30. TPA: NR	NA	NA
Williams et al. (2014) [48]	NA	NA	NA	Short term follow-up: Significant difference between the intervention and control group in proportion of children that used low fat/fat-free milk at home (OR1.39, *p* < 0.05) ES 0.19; and cups of vegetables child consumed at home (∆ + 0.12, p < 0.05) ES 0.12. No significant difference in cups of fruit child consumed at home (∆ + 0.06, NS) ES 0.04; and cups of fruits and vegetables child consumed at home (∆ + 0.19, NS) ES 0.10 between the intervention and control group. Significant difference between the intervention and control group in no. of days the child helped self/requested vegetable as snack (∆0.34, p < 0.05) ES 0.14. No significant difference between intervention and control group in no. of days the child helped self/requested fruit as snack (∆ + 0.24, NS) ES 0.09; no. of days parent offered vegetable as snack (∆ + 0.25, NS) ES 0.11; and no. of days parent offered fruit as snack (∆0.00, NS) ES 0.
Yin et al. (2014) [49]	Short term follow-up: No significant difference between intervention group and control group in BMI z-score (∆-0.09, *p* < 0.09) ES − 0.04.	Short term follow-up: Significant difference between the intervention and control group in gross motor development (∆1.15, p < 0.001) ES 0.03 A significantly higher level of active play in the intervention group compared to the control group (data not available).	NA	Short term follow-up: Significantly more fruit and vegetables consumption in the intervention group (0.19 serving, p < 0.05) and low-fat milk (0.06 serving, *p* < 0.006) than in the control group. No reporting on grain products. No significant change in meat consumption.
Zhou et al. (2014) [50]	Short term follow-up: No significant difference between intervention and control group for BMI (kg/m^2^, ∆0.19, NS) ES 0.10; and BMI z-score (∆0.15, NS) ES 0.10. Significant difference between intervention and control group for % body fat (∆-1.2, *p* = 0.0001) ES − 0.34; fat mass (kg, ∆-0.55, p = 0.0001) ES − 0.61; and muscle mass (kg, ∆ + 0.48, p = 0.0001) ES 0.32.	Short term follow-up: Significant difference between the intervention and control group in 20 m agility run (seconds, ∆-0.74, p = 0.0001) ES − 0.39; broad jump (cm, ∆8.09, p = 0.0001) ES 0.46; tennis ball throw (m, ∆ + 0.52, *p* = 0.006); sit-and-reach (cm, ∆ + 0.88, *p* = 0.03) ES 0.35; balance beam walk (seconds, ∆-2.02, p = 0.0001) ES − 0.15; 20 m crawl (seconds, ∆-3.36, p = 0.0001) ES − 0.55; and 30 m sprint (seconds, ∆-0.45, p = 0.02) ES − 0.21	NA	NA

BMI = Body Mass Index; CPM = Counts Per Minute; HS + POPS = Head Start + Preschool Obesity Prevention Series; IYS = Incredible Years Series; KiMo = Kindergarten Mobile; LPA = Light Physical Activity; MVPA = Moderato-to-Vigorous-Physical-Activity; NA = Not Applicable; NF-P = Nursery Fit-Participated; NF-NP=Nursery Fit-Not Participated; NR = Not Reported; PA = physical activity; TPA = total Physical activity

Effect sizes are only provided for studies and outcomes for which effect sizes could be calculated. The positive or negative indicator shows the direction of effect. Depending on the outcome this favours the intervention group or the control group

One study did not report the BMI or BMI z-score, but reported non-significant differences between the groups on weight and height scores (standardized) [54]. For two studies, no conclusions on BMI or BMI z-scores could be drawn because they were not reported [58] or the data were insufficient (reporting on the whole group instead of the intervention and control groups separately) [39].

Four studies reported additional long-term follow-up measurement. Two of them reported no differences between the intervention and control group [37, 43]. The other two reported unfavourable effects for the intervention group at the long-term follow-up, although they were not significant [44, 46]. The available effect sizes for the long-term follow-up were small [44, 46].

### Effects on physical activity and sedentary behaviour outcomes

With regard to PA outcomes, eleven out of fifteen studies (73.3%) found favourable effects on at least one of the outcomes [38–41, 45–47, 49, 50, 52, 58] (Table 4). Of these studies, three found positive effects on all PA outcomes measured [39, 49, 50], and six found mixed effects [38, 40, 45, 46, 52, 58]. The majority (66.7%) of the significant effects were found for motor development outcomes [40, 45, 46, 49, 50, 58]. The effects found by Lumeng et al. (2017) and Story et al. (2012) were all non-significant [41, 47]. The effect sizes of the favourable results were large [58], moderate [40, 50, 58], and small [38–41, 45, 49, 50]. For two studies [47, 52] effect sizes could not be determined. Two studies found effects that were unfavourable for the intervention group [43, 44]. These results had small effect sizes (non-significant), except for Nyberg et al. (2015) on ‘child taken to activity in the last week’, which had a moderate effect size [43] and was non-significant. One study found no effect on all PA outcomes [51]. One study reported no significant differences for PA outcomes, but did not show data [42].

Three studies had a long-term follow-up of PA outcomes [43, 44, 46]. Roth et al. (2015) found mixed long-term effects of PA outcomes. Some of their outcomes were also unfavourable for the intervention group, but not significant [46]. The two other studies had non-significant unfavourable results, except for MVPA in the study of Nyberg et al. (2015), which was favourable for the intervention group [43, 44]. All long-term effect sizes were small.

Sedentary behaviour was operationalised as time spent in SB or as screen time/media use. Eight out of nine (88.9%) studies found favourable effects of the intervention on at least one SB outcome [38, 39, 41–45, 55]. Five of them found positive effects on all SB outcomes [38, 39, 42, 45, 55]. Of the effective studies, three found effects on screen time/media use [39, 42, 45] and two on time in SB [38, 55]. The available effect sizes of the effective studies were moderate [39] or small [38, 45]. Three studies also reported unfavourable effects for the intervention group on SB outcomes [41, 43, 44]. These results all had small effect sizes and were not significant. One study did not show any effect of the intervention on SB [51].

Two studies performed an additional long-term follow-up [43, 44]. Nyberg et al. (2016) found mixed effects in the long-term with a significant difference in time in SB, with a small effect size [44]. Nyberg et al. (2015) found favourable effects for the intervention group on both SB outcomes in the long-term [43]. These results had a small effect size and were not significant.

### Effects on nutrition behaviour outcomes

All studies reporting on NB outcomes reported favourable results for the intervention group for at least one of the NB outcome [39, 41–45, 47–49, 53–55, 57] (Table 4). Three studies found positive effects on all NB-related outcomes [45, 53, 55]. One study described positive effects, but no conclusions on significance could be made based on the available information [42]. Eight studies found mixed effects [39, 41, 43, 44, 47–49, 54]. Effects were seen in a great variety of NB outcomes, such as fruit and vegetable consumption, junk food consumption, sugar sweetened beverages (SSB) intake, breakfast patterns [39, 41, 47, 53, 55], nutrients in school menus [47], or percentage of healthy eaters [45]. Within these mixed effects, some studies found unfavourable results for the intervention group for some outcomes [39, 41, 43, 44, 47, 57]. They were all non-significant. Effect sizes were available for five studies (38.5%) [41, 43, 44, 47, 48]. One study found large and moderate effect sizes in changes in nutrients from school menus [47]. The studies by Nyberg et al. (2015, 2016) showed large, moderate, and small effect sizes [43, 44]. All other effects on the NB-related outcomes were small [41, 47, 48].

Three studies had an additional long-term follow-up measurement of NB [39, 43, 44]. They all showed favourable results for the intervention group for at least one of the outcomes. One study showed mixed effects [39], and the other two studies showed no significant long-term effects [43, 44]. Some of these non-significant effects were unfavourable for the intervention group. Long-term effect sizes of these two studies on the different NB outcomes were large, moderate and small.

### Synthesizing intervention components with effects

From a narrative synthesis of the effects with the intervention components, two types of patterns emerged. First, better integrated interventions (targeting multiple types of environments) seemed to be related to intervention effectiveness. In particular, incorporating policy changes in addition to changes in the physical and sociocultural environments appeared to increase the likelihood of effects occurring [42, 48, 50, 55]. For example, Zhou et al. (2014) formulated PA policy as part of the intervention and found significant differences in the PA outcomes [50]. In the interventions of Natale, Lopez-Mitnik et al. (2014) and Natale, Messiah et al. (2014), policy was formulated on various EBRBs, and they found significant differences between the intervention and control groups for SB and NB [42, 55]. One intervention focused on policy on NB, but did not report on this outcome and did not find effects on PA [52, 56].

The second pattern that emerged concerned the level of parental involvement, which seemed to be positively related to the intervention effectiveness. For example, an intervention adopting a participatory design, i.e. actively involving parents in the intervention development, showed effects on PA and SB [38]. An intervention using parent-delivered activities found effects on PA [49], and interventions using family activities for both parents and children found effects on various EBRBs [39, 50, 53, 54]. These interventions were found to be more effective than interventions focusing predominantly on parental education [41, 43, 44, 51, 58].

## Discussion

**Fig. 2:**
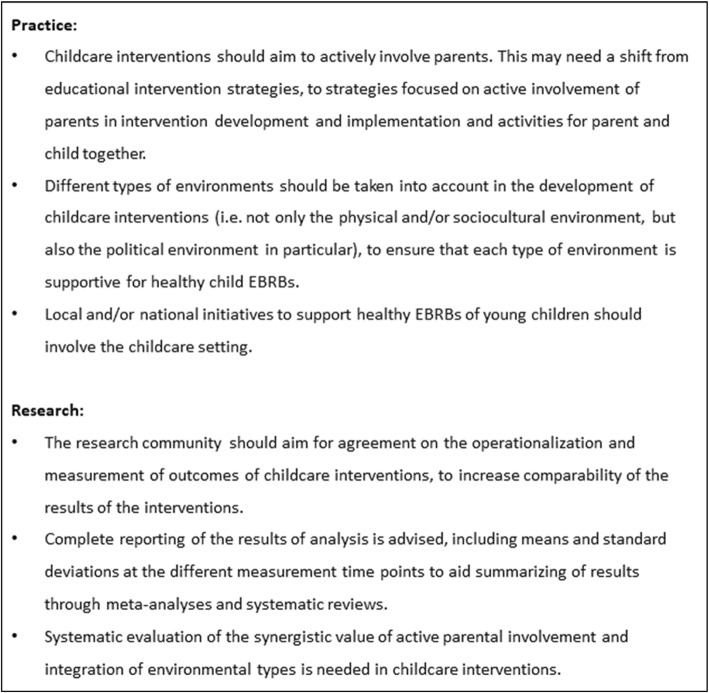
Key recommendations from this systematic review

The aim of this systematic review was to evaluate the effectiveness of childcare-based interventions with direct parental involvement on weight status and EBRBs in children aged 2–5 years old. A total of 22 studies describing 17 interventions was included. These studies showed promising effectiveness with predominantly favourable results for the intervention group on at least one of the measured outcomes. However, there were studies that also showed unfavourable results. The effect sizes related to these results were for a great majority small, with a few moderate and large effect sizes. Only a small number of studies showed statistically significant differences between the intervention and control group, in particular on weight-related outcomes. Figure 2 shows the key recommendations that emerged from this review and that will be explained further here.

The level of parental involvement appeared to positively impact the intervention effectiveness. Interventions that used strategies to actively involve parents through participatory intervention designs, parent-delivered activities, or family activities including both parents and children appeared to have a higher likelihood of success in influencing the children’s EBRBs. A recent qualitative study emphasised the preference of parents to spend quality family time and have fun with the family through participating in such interventions [59]. Some studies indicated possible ceiling effects on health-related beliefs (parents usually know what is healthy), indicating there may be little to be gained from solely educational interventions [59, 60]. This may explain the limited effectiveness of the interventions in this systematic review that focused mainly on health education for parents. An important consideration in interventions using parental involvement may be selection bias. Some parental characteristics are associated with participation in interventions, such as high SES and two-parent families [61]. Cognitive beliefs may influence participation, for example, realising that their child is at risk for a certain behaviour [61, 62]. These factors may also be applicable to health-promoting childcare interventions, resulting in the participation of parents who may be more engaged with the topic. This may influence the effectiveness of these interventions. Reaching and involving parents is a major challenge in interventions aimed at involving parents [63]. Many practical considerations exert important influences on the parents’ ability to participate in interventions [59, 63]. Nevertheless, the high reach of parents may be a precondition to increase intervention effectiveness. In this systematic review it appeared that studies reporting high reach (> 80%) were more likely to have positive results [39, 43, 45, 49, 50]. Three of them used active parental involvement strategies [39, 49, 50]. This might be an indication that parents are more willing to participate in these types of interventions. As the data in this review are not conclusive on reach, there is still a lot to be learned about how to reach parents, what strategies to use in interventions, and how to increase the level of parental involvement, in order to improve health-promoting interventions for young children.

Better integrated interventions, including the political environment, appeared to be related to increased effectiveness. Policies may function as the basis or backbone of intervention strategies and be an important enabler for determinants related to behaviour [64, 65]. For example, promoting water consumption in the childcare setting can be arranged by educating childcare workers and parents and providing a water tap. However, it may become part of common practice and result in more sustainable change if a supporting policy is formulated. This may entail, for example, stating that the serving of SSB is no longer allowed and parents are no longer allowed to bring SSB from home. The findings related to the level of parental involvement and the integration of the types of environment should be interpreted with caution, since they are based on a narrative synthesis of the interventions. A systematic assessment of effective intervention elements is needed to confirm these results.

Factors in all types of environments influence children’s EBRBs [65–68]. It is thus important to take into account the different environmental types. As the political and economic environments have been underrepresented in the interventions included in this systematic review, increased attention should be paid to them by intervention developers. Improving our understanding of the interdependence between the environmental types (e.g. how is the sociocultural environment influenced by the political environment) may help in designing interventions that fit best within their real-life setting and can have a greater impact.

In line with previous reviews, limited evidence was found for effectiveness on weight status outcomes, while more indications were found for effectiveness on behavioural outcomes [29–31]. Interventions thus appear to be more effective in changing behaviour which they directly target. Weight status is changed through the child’s behaviour and therefore more distal and more difficult to change. Time may be an important factor in determining intervention effectiveness on weight status outcomes because behavioural changes need time to manifest as weight changes. In line with this, longer interventions and longer follow-up time resulted in increased odds of effectiveness on weight status [45, 47, 57]. Moreover, interventions showing an effect on weight status also showed effects on one or more behaviour-related outcomes [39, 40, 45, 47, 55, 57]. These effective interventions on weight status all aimed at multiple EBRBs. This emphasizes the importance of not targeting single EBRBs in isolation, but combining them in interventions. This is also supported by research showing the clustering of EBRBs in young children [69] and a recent intervention study showing stronger effects of a comprehensive intervention approach compared to the promotion of physical activity in isolation [70].

Regarding the PA outcomes, most effects were seen on motor skill development. Fundamental motor skills (FMS) are the basis for an active life as children become able to perform activities and enjoy being physically active. This can help them to maintain an active lifestyle throughout their lives [71, 72]. It may be more important to aim interventions at FMS rather than physical intensity measures at this age. A majority of the interventions showing this positive effect on FMS provided play materials as part of the changes in the physical environment [45, 49, 50, 58]. This may suggest that this intervention strategy fits better with effects on FMS.

The NB outcomes were operationalised in many different ways: varying from intake at school and at home, to intake on product level and on nutrient level. Most of the outcomes were subjectively measured by parental self-report. These factors made it difficult to draw conclusions on the effectiveness on NB outcomes. The magnitude of the effects for all outcomes was moderate or small, with some exceptions. However, in the end, all the small effect sizes on different behavioural outcomes, day in and day out, may add up to substantial behavioural change.

Although intervention effectiveness on behavioural outcomes was promising, it may still be considered limited, for example when compared with primary school-based interventions (except for NB outcomes) [33]. Context-related factors may explain this difference in effectiveness. Attention paid to healthy EBRBs in young children has only recently started to grow. This lack of tradition and culture of health promotion in the childcare setting is reflected in the studies included in this review, with the oldest intervention dating from 2001. A longer tradition of promoting healthy EBRBs may facilitate a more positive tendency and greater readiness for intervention implementation, which may result in increased effectiveness. In addition, context-related factors such as local and national health-promoting initiatives have focused mainly on primary school-aged children and older up till now, while these new initiatives aimed at younger children may be very supportive of change [73, 74]. It is important to take into account such context-related factors in intervention development and implementation, as they may be crucial in understanding effectiveness [75].

### Limitations of the included studies

There was great heterogeneity between the included studies regarding operationalisation and measurement of outcome measures. This hindered our ability to perform a meta-analysis of the effects. In addition, comparability of the effects of individual interventions included in this review is limited. Another limitation is the methodological quality of the included studies, as only three studies were rated as strong. However, those three studies were not more effective compared to the other studies. This may be explained by the focus of the quality instrument on internal validity (e.g. study design and randomization, blinding, and dropout rates). These may be aspects that cannot always be taken into account in ‘real-life’ intervention studies.

### Strengths and limitations of the review

This review adds to our knowledge on intervention effectiveness in the childcare setting by specifically looking at direct parental involvement. We tried to explain intervention effects by looking at the different types of environments targeted using the ANGELO framework [11]. The strengths of this review are the use of the EPHPP tool, which is a validated instrument to assess study quality, and thus reflect the risk of bias, for intervention studies [36]; the use of the PRISMA statement for reporting of the systematic review [76]; and calculation of the effect sizes to increase comparability between the studies.

There are some limitations to this systematic review. Although four databases were used to conduct the literature search, only studies written in English were included, which may have resulted in selection bias. We did not extend our literature search to find unpublished work, which may have resulted in publication bias. Results and conclusions of this review may need to be considered with caution due to the mostly weak methodological quality of the included studies. Further, the synthesis of intervention components and effects was based on narrative synthesis and needs further research.

### Recommendations

There is a sound theoretical foundation to incorporate parental involvement in childcare-based interventions [29, 30]. Behavioural outcomes such as children’s EBRBs and intermediaries’ behaviours are more likely to be changed by these types of interventions. Increased attention paid to operationalization and continuity in these outcomes between studies will improve the comparability of intervention programs.

Knowledge also needs to be gained on how to reach parents, what type of strategies to use for parental involvement, and the optimal level of parental involvement. This knowledge could be essential in improving the effectiveness of childcare-based intervention programs. With regard to reporting on intervention results, improvements could be made in the detail of reporting on study design and results (e.g. means and standard deviations). This will enable a better judgement of the study quality and calculation of the effect sizes. A systematic evaluation to determine effective intervention elements may be needed.

We recommend that intervention developers take into account all different types of environments and look beyond the physical and sociocultural environment when designing health-promoting programmes in the childcare setting. In particular, policy changes may function as a necessary additional element in order to achieve sustained effects. We also recommend taking a comprehensive approach (including different EBRBs) and taking into account the clustering of EBRBs. Recognizing the complexity of childhood overweight and obesity in intervention development may be indispensable for intervention effectiveness. We recommend looking for alternative ways of involving parents besides just educational strategies. Formative research may support intervention development by shedding light on influential factors from different types of environments and their interdependence, and will aid in increasing intervention fit with the setting.

## Conclusion

Childcare-based interventions with direct parental involvement show promising effects on improving young children’s EBRBs. However, the evidence is limited, especially for weight-related outcomes. More integration of different types of environment, as well as a more active level of parental involvement, might be factors that influence intervention effects on children’s EBRBs. Taking these factors into account in intervention development may advance the field of childcare-based health promotion towards more effectively and sustainably changing children’s EBRBs.

## Supplementary information

**Additional file 1:** **Table S1.** Search strategy Pubmed.

## Data Availability

All data generated or analyzed during this study are included in this published article.

## Abbreviations

ANGELO: Analysis Grid for Environments Linked to Obesity
BMI: Body Mass Index
EBRBs: Energy Balance-Related Behaviours
FMS: Fundamental Motor Skills
NB: Nutrition Behaviour
PA: Physical Activity
PRISMA: Preferred Reporting Items for Systematic Reviews and Meta-Analyses
RCT: Randomized Controlled Trial
SB: Sedentary Behaviour

## References

[1] Wabitsch M, Moss A, Kromeyer-Hauschild K (2014). Unexpected plateauing of childhood obesity rates in developed countries. BMC Med.

[2] De Kroon MLA, Renders CM, Van Wouwe JP, Van Buuren S, Hirasing RA (2010). The Terneuzen birth cohort: BMI changes between 2 and 6 years correlate strongest with adult overweight. PLoS One.

[3] Cunningham SA, Kramer MR, Narayan KM (2014). Incidence of childhood obesity in the United States. N Engl J Med.

[4] Kremers SP, Visscher TL, Seidell JC, van Mechelen W, Brug J (2005). Cognitive determinants of energy balance-related behaviours: measurement issues. Sports Med.

[5] Rennie KL, Johnson L, Jebb SA (2005). Behavioural determinants of obesity. Best Pract Res Clin Endocrinol Metab.

[6] Gubbels JS, Kremers SP, Goldbohm RA, Stafleu A, Thijs C (2012). Energy balance-related behavioural patterns in 5-year-old children and the longitudinal association with weight status development in early childhood. Public Health Nutr.

[7] Telama R, Yang X, Leskinen E, Kankaanpaa A, Hirvensalo M, Tammelin T (2014). Tracking of physical activity from early childhood through youth into adulthood. Med Sci Sports Exerc.

[8] Fernandez-Jimenez R, Al-Kazaz M, Jaslow R, Carvajal I, Fuster V (2018). Children present a window of opportunity for promoting health: JACC review topic of the week. J Am Coll Cardiol.

[9] Kremers SPJ, de Bruijn GJ, Visscher TL, van Mechelen W, de Vries NK, Brug J (2006). Environmental influences on energy balance-related behaviors: a dual-process view. Int J Behav Nutr Phys Act.

[10] Gubbels JS, Van Kann DH, de Vries NK, Thijs C, Kremers SP (2014). The next step in health behavior research: the need for ecological moderation analyses - an application to diet and physical activity at childcare. Int J Behav Nutr Phys Act.

[11] Swinburn B, Egger G, Raza F (1999). Dissecting obesogenic environments: the development and application of a framework for identifying and prioritizing environmental interventions for obesity. Prev Med.

[12] Eurostat. Eurostat News Release. Childcare in the EU in 2006. A quarter of children aged less than three in formal childcare Eurostat Press Office; 2008.

[13] Laughlin L (2013). Who's minding the kids? Childcare arrangements: spring 2011.

[14] Perrin EM, Howard JB, Ward DS (2016). In the absence of clear causation, casting a wider net for prevention. Pediatrics.

[15] Geoffroy MC, Power C, Touchette E, Dubois L, Boivin M, Seguin JR (2013). J Pediatr.

[16] Alberdi G, McNamara AE, Lindsay KL, Scully HA, Horan MH, Gibney ER (2016). The association between childcare and risk of childhood overweight and obesity in children aged 5 years and under: a systematic review. Eur J Pediatr.

[17] Gubbels JS, Kremers SP, Stafleu A, Dagnelie PC, de Vries NK, van Buuren S (2010). Child-care use and the association with body mass index and overweight in children from 7 months to 2 years of age. Int J Obes.

[18] Ward S, Belanger M, Donovan D, Carrier N (2015). Systematic review of the relationship between childcare educators' practices and preschoolers' physical activity and eating behaviours. Obes Rev.

[19] Escalante Y, Garcia-Hermoso A, Backx K, Saavedra JM (2014). Playground designs to increase physical activity levels during school recess: a systematic review. Health Educ Behav.

[20] Temple M, Robinson JC (2014). A systematic review of interventions to promote physical activity in the preschool setting. J Spec Pediatr Nurs.

[21] Gubbels JS, Kremers SP, Stafleu A, de Vries SI, Goldbohm RA, Dagnelie PC (2011). Association between parenting practices and children's dietary intake, activity behavior and development of body mass index: the KOALA birth cohort study. Int J Behav Nutr Phys Act.

[22] Sleddens EF, Gerards SM, Thijs C, de Vries NK, Kremers SP (2011). General parenting, childhood overweight and obesity-inducing behaviors: a review. Int J Pediatr Obes.

[23] Hendrie GA, Coveney J, Cox DN (2012). Defining the complexity of childhood obesity and related behaviours within the family environment using structural equation modelling. Public Health Nutr.

[24] Bradley RH, Evans GW, Wachs TD (2010). From home to day care: chaos in the family/child-care mesosystem. Chaos and its influence on children’s development an ecological perspective.

[25] Gubbels JS, Stessen K, van de Kolk I, de Vries NK, Thijs C, Kremers SPJ (2018). Energy balance-related parenting and child-care practices: the importance of meso-system consistency. PLoS One.

[26] Kader M, Sundblom E, Elinder LS (2015). Effectiveness of universal parental support interventions addressing children's dietary habits, physical activity and bodyweight: a systematic review. Prev Med.

[27] Sisson SB, Krampe M, Anundson K, Castle S (2016). Obesity prevention and obesogenic behavior interventions in child care: a systematic review. Prev Med.

[28] Waters E, de Silva-Sanigorski A, Burford BJ, Brown T, Campbell KJ, Gao Y, et al. Interventions for preventing obesity in children. Cochrane Database Syst Rev. 2011;12. 10.1002/14651858.CD001871.pub3 22161367

[29] Ling J, Robbins LB, Wen F (2016). Interventions to prevent and manage overweight or obesity in preschool children: a systematic review. Int J Nurs Stud.

[30] Ward DS, Welker E, Choate A, Henderson KE, Lott M, Tovar A (2017). Strength of obesity prevention interventions in early care and education settings: a systematic review. Prev Med.

[31] Morris H, Skouteris H, Edwards S, Rutherford L (2015). Obesity prevention interventions in early childhood education and care settings with parental involvement: a systematic review. Early Child Dev Care.

[32] Hingle MD, O'Connor TM, Dave JM, Baranowski T (2010). Parental involvement in interventions to improve child dietary intake: a systematic review. Prev Med.

[33] Verjans-Janssen SRB, van de Kolk I, Van Kann DHH, Kremers SPJ, Gerards SMPL (2018). Effectiveness of school-based physical activity and nutrition interventions with direct parental involvement on children’s BMI and energy balance-related behaviors – a systematic review. PLoS One.

[34] Cohen J (1988). Statistical power analysis for the behavioral sciences.

[35] Lipsey MW (1990). Design sensitivity: statistical power for experimental research: sage.

[36] Thomas BH, Ciliska D, Dobbins M, Micucci S (2004). A process for systematically reviewing the literature: providing the research evidence for public health nursing interventions. Worldviews Evid-Based Nurs.

[37] Cespedes J, Briceno G, Farkouh ME, Vedanthan R, Baxter J, Leal M (2013). Targeting preschool children to promote cardiovascular health: cluster randomized trial. Am J Med.

[38] De Bock F, Genser B, Raat H, Fischer JE, Renz-Polster H (2013). A participatory physical activity intervention in preschools: a cluster randomized controlled trial. Am J Prev Med.

[39] Kaufman-Shriqui V, Fraser D, Friger M, Geva D, Bilenko N, Vardi H (2016). Effect of a school-based intervention on nutritional knowledge and habits of low-socioeconomic school children in Israel: a cluster-randomized controlled trial. Nutrients.

[40] Klein D, Manz K, Ferrari N, Struder H, Graf C (2015). Effects of health promotion projects in preschools on body mass index and motor abilities. J Sports Med Phys Fitness.

[41] Lumeng JC, Miller AL, Horodynski MA, Brophy-Herb HE, Contreras D, Lee H (2017). Improving self-regulation for obesity prevention in head start: a randomized controlled trial. Pediatrics.

[42] Natale RA, Lopez-Mitnik G, Uhlhorn SB, Asfour L, Messiah SE (2014). Effect of a child care center-based obesity prevention program on body mass index and nutrition practices among preschool-aged children. Health Promot Pract.

[43] Nyberg G, Sundblom E, Norman A, Bohman B, Hagberg J, Elinder LS (2015). Effectiveness of a universal parental support programme to promote healthy dietary habits and physical activity and to prevent overweight and obesity in 6-year-old children: the healthy school start study, a cluster-randomised controlled trial. PLoS One.

[44] Nyberg G, Norman A, Sundblom E, Zeebari Z, Elinder LS (2016). Effectiveness of a universal parental support programme to promote health behaviours and prevent overweight and obesity in 6-year-old children in disadvantaged areas, the healthy school start study II, a cluster-randomised controlled trial. Int J Behav Nutr Phys Act.

[45] Puder JJ, Marques-Vidal P, Schindler C, Zahner L, Niederer I, Burgi F (2011). Effect of multidimensional lifestyle intervention on fitness and adiposity in predominantly migrant preschool children (Ballabeina): cluster randomised controlled trial. BMJ..

[46] Roth K, Kriemler S, Lehmacher W, Ruf KC, Graf C, Hebestreit H (2015). Effects of a physical activity intervention in preschool children. Med Sci Sports Exerc.

[47] Story M, Hannan PJ, Fulkerson JA, Rock BH, Smyth M, Arcan C (2012). Bright start: description and main outcomes from a group-randomized obesity prevention trial in American Indian children. Obesity (Silver Spring).

[48] Williams PA, Cates SC, Blitstein JL, Hersey J, Gabor V, Ball M (2014). Nutrition-education program improves preschoolers' at-home diet: a group randomized trial. J Acad Nutr Diet.

[49] Yin Z, Parra-Medina D, Cordova A, He M, Trummer V, Sosa E (2012). Miranos! Look at us, we are healthy! An environmental approach to early childhood obesity prevention. Child Obes.

[50] Zhou Z, Ren H, Yin Z, Wang L, Wang K (2014). A policy-driven multifaceted approach for early childhood physical fitness promotion: impacts on body composition and physical fitness in young Chinese children. BMC Pediatr.

[51] Adamo KB, Wasenius NS, Grattan KP, Harvey ALJ, Naylor PJ, Barrowman NJ (2017). Effects of a preschool intervention on physical activity and body composition. J pediatrics.

[52] Cruz TH, Davis SM, Myers OB, O'Donald ER, Sanders SG, Sheche JN (2016). Effects of an obesity prevention intervention on physical activity among preschool children: the CHILE study. Health Promot Pract.

[53] Gao Y, Cai C, Li J, Sun W (2016). Nutritional intervention and breakfast behavior of kindergartens. Iran J Public Health.

[54] Hu C, Ye D, Li Y, Huang Y, Li L, Gao Y (2010). Evaluation of a kindergarten-based nutrition education intervention for pre-school children in China. Public Health Nutr.

[55] Natale RA, Messiah SE, Asfour L, Uhlhorn SB, Delamater A, Arheart KL (2014). Role modeling as an early childhood obesity prevention strategy: effect of parents and teachers on preschool children’s healthy lifestyle habits. J Dev Behav Pediatr.

[56] Davis SM, Myers OB, Cruz TH, Morshed AB, Canaca GF, Keane PC (2016). CHILE: outcomes of a group randomized controlled trial of an intervention to prevent obesity in preschool Hispanic and American Indian children. Prev Med.

[57] Natale RA, Messiah SE, Asfour LS, Uhlhorn SB, Englebert NE, Arheart KL (2017). Obesity prevention program in childcare centers: two-year follow-up. Am J Health Promot.

[58] Wasenius NS, Grattan KP, Harvey ALJ, Naylor PJ, Goldfield GS, Adamo KB (2018). The effect of a physical activity intervention on preschoolers' fundamental motor skills - a cluster RCT. J Sci Med Sport.

[59] Rhodes RE, Lim C. Promoting parent and child physical activity together: elicitation of potential intervention targets and preferences. Health Educ Behav. 2017;1090198117704266. 10.1177/1090198117704266 28415853

[60] Martin-Biggers J, Spaccarotella K, Hongu N, Alleman G, Worobey J, Byrd-Bredbenner C (2015). Translating it into real life: a qualitative study of the cognitions, barriers and supports for key obesogenic behaviors of parents of preschoolers. BMC Public Health.

[61] Spoth R, Redmond C (2000). Research on family engagement in preventive interventions: toward improved use of scientific findings in primary prevention practice. J Prim Prev.

[62] Pettersson C, Linden-Bostrom M, Eriksson C (2009). Reasons for non-participation in a parental program concerning underage drinking: a mixed-method study. BMC Public Health.

[63] Heath SM, Wigley CA, Hogben JH, Fletcher J, Collins P, Boyle GL (2018). Patterns in participation: factors influencing parent attendance at two, Centre-based early childhood interventions. J Child Fam Stud.

[64] Hendriks AM, Habraken J, Jansen MW, Gubbels JS, De Vries NK, van Oers H (2014). 'Are we there yet?' - operationalizing the concept of integrated public health policies. Health Policy.

[65] van de Kolk I, Goossens AJM, Gerards S, Kremers SPJ, Manders RMP, Gubbels JS (2018). Healthy Nutrition and Physical Activity in Childcare: Views from Childcare Managers, Childcare Workers and Parents on Influential Factors. Int J Environ Res Public Health.

[66] Mazarello Paes V, Ong KK, Lakshman R (2015). Factors influencing obesogenic dietary intake in young children (0-6 years): systematic review of qualitative evidence. BMJ Open.

[67] Tucker P, van Zandvoort MM, Burke SM, Irwin JD (2011). The influence of parents and the home environment on preschoolers' physical activity behaviours: a qualitative investigation of childcare providers' perspectives. BMC Public Health.

[68] Wilke S, Opdenakker C, Kremers SP, Gubbels JS (2013). Factors influencing childcare workers’ promotion of physical activity in children aged 0–4 years: a qualitative study. Early Years.

[69] Gubbels JS, Kremers SP, Stafleu A, Dagnelie PC, de Vries SI, de Vries NK (2009). Clustering of dietary intake and sedentary behavior in 2-year-old children. J Pediatr.

[70] Bartelink NHM, van Assema P, Kremers SPJ, Savelberg HHCM, Oosterhoff M, Willeboordse M (2019). One- and two-year effects of the healthy primary School of the Future on Children’s dietary and physical activity Behaviours: a quasi-experimental study. Nutrients..

[71] Lloyd M, Saunders TJ, Bremer E, Tremblay MS (2014). Long-term importance of fundamental motor skills: a 20-year follow-up study. Adapt Phys Act Q.

[72] Loprinzi PD, Davis RE, Fu YC (2015). Early motor skill competence as a mediator of child and adult physical activity. Prev Med Rep.

[73] Borys JM, Le Bodo Y, Jebb SA, Seidell JC, Summerbell C, Richard D (2012). EPODE approach for childhood obesity prevention: methods, progress and international development. Obes Rev.

[74] Jongeren Op Gezond Gewicht. JOGG Programme n.d. Available from: https://jongerenopgezondgewicht.nl/jogg-aanpak. .

[75] Hawe P, Shiell A, Riley T (2009). Theorising interventions as events in systems. Am J Community Psychol.

[76] Moher D, Liberati A, Tetzlaff J, Altman DG (2009). Preferred reporting items for systematic reviews and meta-analyses: the PRISMA statement. BMJ..

